# Pairwise quantum criteria and teleportation in a spin square complex

**DOI:** 10.1038/s41598-022-10248-2

**Published:** 2022-04-18

**Authors:** Fadwa Benabdallah, Saeed Haddadi, Hamid Arian Zad, Mohammad Reza Pourkarimi, Mohammed Daoud, Nerses Ananikian

**Affiliations:** 1 LPHE-Modeling and Simulation, Faculty of Sciences, Mohammed V University, Rabat, Morocco; 2 Faculty of Physics, Semnan University, P.O.Box 35195-363, Semnan, Iran; 3 Saeed’s Quantum Information Group, P.O.Box 19395-0560, Tehran, Iran; 4 A. I. Alikhanyan National Science Laboratory, 0036 Yerevan, Armenia; 5 Department of Theoretical Physics and Astrophysics, Faculty of Science, P.J. Šafárik University, Park Angelinum 9, 041 54 Košice, Slovak Republic; 6 Department of Physics, Salman Farsi University of Kazerun, Kazerun, Iran; 7 Department of Physics, Faculty of Sciences, University Ibn Tofail, Kenitra, Morocco; 8 Abdus Salam International Centre for Theoretical Physics, Strada Costiera 11, 34151 Trieste, Italy; 9 CANDLE Synchrotron Research Institute, Acharyan 31, 0040 Yerevan, Armenia

**Keywords:** Information theory and computation, Quantum physics

## Abstract

Thermal non-classical correlations quantified by concurrence entanglement, local quantum uncertainty, and quantum coherence in a four-qubit square chain are exactly examined. The influences of the Hamiltonian parameters on the mentioned pairwise quantum criteria and fidelity of teleportation are studied, and the most interesting findings are discussed in detail. It is found that the tuning anisotropy results in enhancing the thermal quantum correlations and coherence as well as average fidelity until achieving maximum values. We persuasively deduce that quantum coherence is a more efficient criterion than that of concurrence and local quantum uncertainty to detect the quantumness of a thermal state.

## Introduction

During the past two decades, various measures of quantum correlations in bipartite and multipartite systems have been proposed, and their properties have been intensively investigated^1–5^. The most potential resource is quantum entanglement^6,7^, which has been considered as the unique form of quantum correlations to enhance the quantum information technology at the time. Indeed, some separable quantum states may also perform better than their classical counterparts for certain quantum tasks^8,9^. Nevertheless, according to various studies^10–14^, quantum correlations can not only be limited to quantum entanglement. Inspired by the Wigner-Yanase skew information^15^, the local quantum uncertainty (LQU) has been introduced by Girolami et al.^16^ as a discord-like quantifier of non-classical correlations in multipartite systems^17^. It quantifies the uncertainty which can arise in a given quantum state due to its noncommutativity with the measured local observable^18^. The LQU is defined as a minimum of the skew information and a closed mathematical expression is available for any bipartite system^16^. Furthermore, beyond its importance as a quantum correlation quantifier, LQU is relatively associated with the notion of quantum Fisher information^19–22^, which makes it an easy-to-access key for quantum metrology protocols^16^. Put together, the Wigner-Yanase skew information is also connected with quantum coherence^23–25^. This approach is one of the subjects of interest in this paper. By arising from quantum state superposition, quantum coherence is one of the central concepts for quantum information processing^26,27^. It has been widely used as an important resource for quantum technology^28–32^, with further relevant applications including quantum optics^26^, quantum information science^33,34^, thermodynamics^35,36^, and so forth^37–39^. Besides, the quantum coherence could be pleasantly linked to quantum entanglement and entropic uncertainty relations^40–47^, and discord-like correlations as well^48^. Motivated by this connection, a wide variety of quantum coherence measurements have been proposed, and their properties have been investigated in detail over the years^25,48^. For instance, Baumgratz et al.^25^ formulated a rigorous resource framework for the quantification of quantum coherence. They proposed the relative entropy of coherence and the intuitive $l_{1}-$norm quantum coherence as proper quantifiers of quantum coherence, which take the form of easy to evaluate analytical expressions. Moreover, Hu et al.^48^ examined the discord-like quantum correlations and quantum coherence measures for bipartite and multipartite systems, and their relationship in various settings. The authors also provided a full review of the resource theory about the discord-like quantum correlations and quantum coherence, which are defined based on the different distance measures of states.

**Figure 1:**
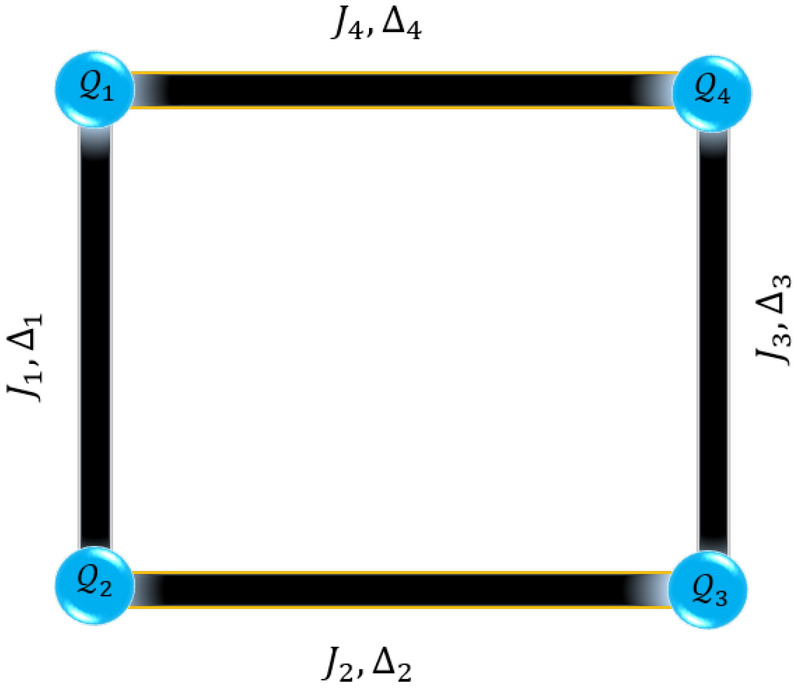
Schematic structure of a four-qubit ($Q_{1}$, $Q_{2}$, $Q_{3}$, and $Q_{4}$) square complex on the Heisenberg XXZ model with the corresponding exchange anisotropy and spin interactions.

The exactly solvable spin chains provide notable cornerstones of the quantum theory of magnetism^49–52^. The 1-D ferrimagnetically Heisenberg spin chains have attracted a great deal of attention, for the reason that they naturally bear strong magnetic properties and demonstrate some zero-temperature phase transitions between intriguing ground states that properly coincide with the quantized magnetization plateaus in respective magnetization curves^53–55^. The structural and magnetic properties of the ferromagnetically coupled tetranuclear copper $\text {Cu}_{4}^{\text {II}}$ square complex have been experimentally investigated in Ref.^56^. The spin arrangement of this special complex in the crystal lattice leads to the formation of a square structure. Motivated by the considered model and results reported in this reference, in the present paper, we consider a four-qubit square complex to give a detailed investigation of the characteristics of thermal non-classical correlations including bipartite quantum entanglement, LQU, and quantum coherence at finite temperature, as well as, to demonstrate how these quantities behave in such a system at a thermal regime. Difference or similarity between thermal entanglement and other thermal quantum correlation quantifiers will be referred to^57,58^. In this respect, we handle the concurrence, LQU, and the intuitive $l_{1}-$norm quantum coherence for describing the thermal pairwise quantum criteria in a four-qubit square compound on the spin-1/2 Heisenberg XXZ model, under the influence of the external magnetic field, isotropic coupling constant, and exchange anisotropy.

We evaluate the thermal state of the system under consideration by adopting the partitioning scheme, which can be realized by considering the bipartite reduced density matrix whereby one traces over all other systems, leaving effectively only a two-qubit system, which allows us to carry out the calculation of different amounts of quantum correlations related to such a state. Within the comparison framework, the effects of an external magnetic field, inter-chain XX coupling constant and that of the exchange anisotropy in the *z*-direction on the parameter dependence of the aforementioned thermal pairwise quantum correlation measurements are rigorously examined. Finally, we verify the quantum teleportation of two qubits in an arbitrary pure entangled state through the model under consideration in thermal equilibrium as a quantum channel^59–63^. In fact, we investigate the output quantum correlations, coherence, and average fidelity.

This paper is arranged as follows. In "The model Section", we describe the physical model and its eigenstates with the corresponding eigenvalues. Furthermore, the exact solution of the model via the partitioning scheme is obtained in "Pairwise density operator Section". In "Quantum correlations and coherence Section", a brief review concerning the definition of concurrence entanglement, LQU, quantum coherence and their analytical expressions is given. In "Results and discussion Section", the effects of anisotropy, exchange interaction, and external magnetic field on the quantum correlations and coherence are discussed in detail. By using the standard teleportation protocol, we deal with the evaluation of the fidelity, average fidelity, and the non-classical correlations of teleported state or output state in "Quantum teleportation Section". Finally, the concluding remarks are given in "Concluding remarks and outlook Section".

### The model

In this section, let us consider the Hamiltonian of a four-qubit Heisenberg XXZ model as a cluster system, which is under the influence of an external magnetic field (see Fig. 1). The Hamiltonian of the model can be expressed as follows^64,65^

1 $$\begin{aligned} H=\sum _{i}^{4}\left[ -J_{i}(S_{i}^{x}S_{i+1}^{x}+S_{i}^{y}S_{i+1}^{y})+\Delta _{i}S_{i}^{z}S_{i+1}^{z}\right] -\mu _{B}gB\sum _{i}^{4}S_{i}^{z}, \end{aligned}$$

where $J_{i}$ with $i=\{1,2,3,4\}$ describes the strength of the spin interaction, being ferromagnetic when $J_{i}>0$. *B* is the external magnetic field, which is only applied to the *z*-direction, while $\Delta$ is the exchange anisotropy parameter. Moreover, *g* is the Landé *g*-factor with the assumption $g=2$, and $S_{i}^{\alpha }$ ($\alpha =x,y,z$) are the spin-1/2 operators. For convenience, we set $\hbar =1$ and Bohr magneton $\mu _{B}$ was absorbed into a definition of the magnetic field term. Let us notify that for simplicity, in the forthcoming analytical expressions and simulations, we consider $J_{2}=J_{3}=J_{4}=J$ and uniform anisotropy $\Delta _{1}=\Delta _{2}=\Delta _{3}=\Delta _{4}=\Delta$, assuming $J_{1}=1$ as energy unit for all other parameters with $\{B,\;J,\;\Delta ,\;T\}$ being dimensionless parameters. In the next sections, we will assume both pure ferromagnetic interactions ($J>0$) and mixed ferromagnetic-antiferromagnetic interactions between nearest-neighbor spins by adopting $J<0$ that means $J_{2},J_{3},J_{4}<0$ but $J_{1}=1>0$. Regarding previous reports, some real magnetic compounds can be plausibly characterized in terms of our modeled spin-1/2 system^66^.

By straightforward calculations, one can find the eigenvalues and corresponding eigenstates of the mentioned Hamiltonian (1) in terms of the standard basis, as reported in Methods.

### Pairwise density operator

For a system in thermal equilibrium at a temperature *T* (canonical ensemble), the state of the system is given by the density operator $\rho _{ \text {total}}(T)$ $=\exp \left( -\beta H\right) /Z$, with $Z=\mathrm {Tr}\left[ \exp (-\beta H)\right]$ being the partition function of system and $\beta ={ 1/{{k_{B}}T}}$. Hereafter, the Boltzmann’s constant is set to the unit for simplicity, i.e., $k_{B}=1$. Hence, the total density operator $\rho _{\text { total}}\ (T)$ of the described system can be characterized in terms of the eigenstates and eigenvalues of the Hamiltonian (1) as

2 $$\begin{aligned} \rho _{\text {total}}(T)=\frac{1}{Z}\sum \limits _{l=1}^{16}\exp \left( -\beta E_{l}\right) \left| \psi _{l}\right\rangle \left\langle \psi _{l}\right| , \end{aligned}$$

where $E_{l}$ and $\left| \psi _{l}\right\rangle$ are the eigenvalues and eigenstates of the Hamiltonian, respectively, as given in Methods. The whole system can be partitioned by considering the bipartite reduced density matrix of two-qubit $\rho _{ij}(T)=\mathrm {Tr}_{kl}\left[ \rho _{ijkl}(T)\right]$ with $\rho _{ijkl}(T)$=$\rho _{\text {total}}(T)$, which obtained by tracing over all other systems except subsystems or modes *i* and *j*. In total, there are six different density matrices for our considered system. However, we find that the thermal state of $\rho _{12}(T)$ has the highest amount of quantum correlations and coherence. Therefore, from now on, let us consider only this state for analysis. In the standard basis of $\{\left| 00\right\rangle ,\left| 01\right\rangle ,\left| 10\right\rangle ,\left| 11\right\rangle \}$, the reduced density matrix is given by

3 $$\begin{aligned} \rho _{12}(T)\equiv \rho _T = \begin{pmatrix} \rho _{11} &{} . &{} . &{} . \\ . &{} \rho _{22} &{} \rho _{23} &{} . \\ . &{} \rho _{23} &{} \rho _{33} &{} . \\ . &{} . &{} . &{} \rho _{44} \end{pmatrix}, \end{aligned}$$

with the dots which are placed instead of zero entries. Since the components of the above matrix are too long to work with conveniently, let us eschew reporting them here.

### Quantum correlations and coherence

In this section, let us discuss the main results obtained from the theoretical studies of pairwise density matrix and three described quantum criteria such as entaglement concurrence, LQU, and $l_{1}-$norm of quantum coherence.

#### Entaglement concurrence

In order to describe the thermal quantum entanglement in the reduced density matrix $\rho _T$, we employ the most widely accepted measure for a two-qubit system $\rho$ called concurrence $C(\rho )$, which has been described by Wootters^7^. The case $C(\rho )=0$ happens when the system state is separable, whereas $C(\rho )=1$ reveals maximally entangled state. A straightforward definition of concurrence can be expressed as follow

4 $$\begin{aligned} C(\rho ) = \max \{0,\sqrt{\lambda _{1}}-\sqrt{\lambda _{2}}-\sqrt{\lambda _{3}}-\sqrt{\lambda _{4}}\}, \end{aligned}$$

where $\lambda _{i}(i=1,2,3,4)$ are the eigenvalues in the decreasing order of the 4$\times$4 matrix $R=\rho (\sigma _{y}\otimes \sigma _{y})\rho ^{*}(\sigma _{y}\otimes \sigma _{y})$, in which $\rho ^{*}$ and $\sigma _{y}$ are, respectively, the complex conjugate of $\rho$ in the standard basis and the $y-$component Pauli matrix. Ultimately, the concurrence for our thermal state (3) can be achieved by

5 $$\begin{aligned} C(\rho _T ) = 2\max \{0,|\rho _{23}|-\sqrt{\rho _{11} \rho _{44}}\}. \end{aligned}$$

#### Local quantum uncertainty

The LQU was recently proposed as a discord-like measure of quantum correlations based on the principle of skew information^16^. It is written as

6 $$\begin{aligned} \mathscr {U}(\rho )=\min _{K_{\text {A}}}\mathscr {I(}\rho ,K_{\text {A}}\otimes I_{\text {B}}\mathcal {)}, \end{aligned}$$

where $K_{\text {A}}$ is a Hermitian operator (local observable) on the subsystem A admitting a non-degenerate spectrum. $I_{\text {B}}$ being the identity operator acting on the subsystem B, while $\mathscr {I}$ is the Wigner-Yanase skew information associated to the density matrix $\rho$ and defined as^15^

7 $$\begin{aligned} \mathscr {I}(\rho ,K_{\text {A}}\otimes I_{\text {B}})=-\frac{1}{2}\mathrm {Tr}[( \sqrt{\rho },K_{\text {A}}\otimes I_{\text {B}})^{2}]. \end{aligned}$$

Here, $\mathscr {I}$ is non-negative and non-increasing under classical mixing^17,18^. For a $2\otimes d$ (qubit-qudit) bipartite quantum systems^16,18^, the closed-form of the LQU is given by

8 $$\begin{aligned} \mathscr {U}(\rho )=1-\lambda _{\max }(\mathscr {W}_{\text {AB}}\mathcal {)}, \end{aligned}$$

where $\lambda _{\max }$ stands for the largest eigenvalue of the $3\times 3$ matrix $\mathscr {W}_{\text {AB}}$ whose elements are given by^67,68^

9 $$\begin{aligned} (\mathscr {W}_{\text {AB}})_{i j}\equiv \mathrm {Tr}[ \sqrt{\rho }\left( \sigma _{\text {A}}^{i}\otimes I_{\text {B}}\right) \sqrt{\rho }( \sigma _{\text {A}}^{j}\otimes I_{\text {B} }) ], \end{aligned}$$

where $\sigma _{\text {A}}^{i (j)}$ with $i (j)=\{x,y,z\}$ represent the Pauli operators of the subsystem A. The LQU provides a reliable quantifier of quantum correlations and it has a geometrical significance in terms of Hellinger distance^16,18^. It is clear that having the matrix $\mathscr {W}_{\text {AB}}$, one can easily evaluate the LQU for qubit-qudit quantum systems contrarily to quantum discord^17^. This is quite an easy task compared with the complicated minimization process over parameters due to the local measurements^13,16,18^. We notice that for a two-qubit pure state, the LQU coincides with the concurrence and vanishes for classically correlated states. Moreover, it is invariant under local unitary operations^16,18^.

In the Fano-Bloch representation^69,70^, our thermal state $\rho _T$ (3) can be written as follows

10 $$\begin{aligned} \rho _T =\frac{1}{{4}}\sum \limits _{\alpha ,\beta =0}^{{3}}\mathscr {R}{{{ _{\alpha \beta }}}}\left( {\sigma }^{\alpha }{{\otimes {\sigma ^{\beta }}}} \right) , \end{aligned}$$

where $\mathscr {R}{{{_{\alpha \beta }}}}=\mathrm {Tr}\left[ \rho _T (\text { }{ \sigma }^{\alpha }{{\otimes {\sigma ^{\beta })}}}\right]$ are the components of the total correlation tensor occurring in the Fano-Bloch decomposition associated with bipartite density matrix $\rho _T$. The non-vanishing components $\mathscr {R}{{{_{\alpha \beta }}}}$ are given as

11 $$\begin{aligned} \mathscr {R}_{00}= & {} \mathrm {Tr}[\rho _T ]=1,\quad \mathscr {R}_{03}=1-2(\rho _{22}+\rho _{44}),\quad {\mathscr {R}_{11}=\mathscr {R}_{22}=2\rho _{23}}, \nonumber \\ \mathscr {R}_{30}= & {} 1-2(\rho _{33}+\rho _{44}), \quad \mathscr {R}_{33}=1-2(\rho _{22}+\rho _{33}). \end{aligned}$$

Therefore, in terms of the Fano-Bloch components $\mathscr {R}_{\alpha \beta }$ associated with the matrix $\rho _T$, the eigenvalues of $(\mathscr {W}_{\text {AB}})_{ij}$ can be expressed as

12 $$\begin{aligned} \omega _{1}= & {} \omega _{2}=\sqrt{\left( t_{1}+2\sqrt{d_{1}}\right) \left( t_{2}+2\sqrt{d_{2}}\right) }+\frac{1}{4}\frac{\left( \mathscr {R} _{03}^{2}-\mathscr {R}_{30}^{2}\right) }{\sqrt{\left( t_{1}+2\sqrt{d_{1}} \right) \left( t_{2}+2\sqrt{d_{2}}\right) }}, \end{aligned}$$

13 $$\begin{aligned} \omega _{3}= & {} \frac{1}{2}\left[ 1+2\left( \sqrt{d_{1}}+\sqrt{d_{2}} \right) \right] +\frac{1}{8}\left[ \frac{\left( \mathscr {R}_{03}+\mathscr {R} _{30}\right) ^{2}}{ \left( t_{1}+2\sqrt{d_{1}}\right) }+\frac{\left( \mathscr {R}_{03}-\mathscr {R} _{30}\right) ^{2}- 4\mathscr {R}_{11}^{2}}{ \left( t_{2}+2\sqrt{d_{2}}\right) }\right] , \end{aligned}$$

with

14 $$\begin{aligned} t_{1,2}= & {} \frac{1}{2}\left( 1\pm \mathscr {R}_{03}\right) , \end{aligned}$$

15 $$\begin{aligned} d_{1,2}= & {} \frac{1}{16}\left[ \left( 1\pm \mathscr {R} _{33}\right) ^{2}-\left( \mathscr {R}_{30}\pm \mathscr {R}_{03}\right) ^{2}-\left( \mathscr {R}_{11}\mp \mathscr {R}_{22}\right) ^{2}\right] . \end{aligned}$$

Hence, the LQU for the considered thermal state $\rho _T$ is gained from

16 $$\begin{aligned} \mathscr {U}(\rho _T )=1-\max \{\omega _{1}, \omega _{3}\}. \end{aligned}$$

#### Quantum coherence

Quantum coherence is an indisputable physical resource of quantum information processing protocols. Even though quantum coherence in multipartite states is somehow related to quantum correlations, it is a quantum property behind any correlation. Thus, coherence is a quantum resource different from entanglement and discord-like correlations^48^. Regarding the latter, the amount of non-classical correlations is estimated in terms of the coherence, in the sense that coherence of subsystems could act as an upper bound for the quantum discord-like correlation of the total bipartite system.

Herein, we use the intuitive $l_{1}-$norm of coherence measure^25^, defined as the sum of the absolute off-diagonal elements of a quantum state $\rho$ in the reference basis $\{\vert i\rangle \}$. It can be calculated as^71^

17 $$\begin{aligned} C_{l_{1}}\mathcal {(}\rho \mathcal {)=}\sum _{i\ne j}|\left\langle i\right| \rho \left| j\right\rangle |. \end{aligned}$$

Thus, the corresponding $l_{1}-$norm of quantum coherence of the our system described by the reduced thermal state $\rho _T$ (3) is given by

18 $$\begin{aligned} C_{l_{1}}\mathcal {(\rho _T )}=2|\mathcal {\rho }_{23}|. \end{aligned}$$

It has been proved by Streltsov *et al.*^72^ that quantum coherence can be used as a resource for quantum entanglement creation. The $l_{1}-$norm of coherence is a crucial link between various coherence measurements and entanglement^73^. Although, quantum coherence may capture quantumness more than discord-like measures of quantum correlations, the later is exactly basis-independent measure of the former^25,74^.

**Figure 2:**
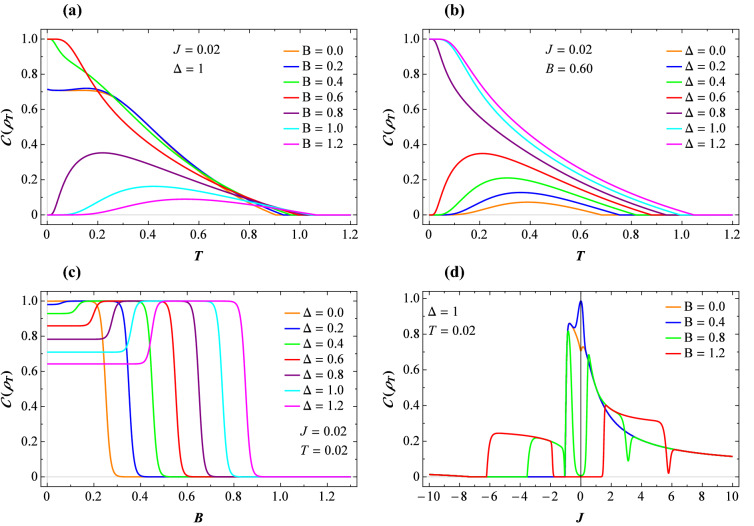
(**a**) Concurrence versus temperature for several fixed values of the magnetic field, taking $J=0.02$ and $\Delta =1$. (**b**) Concurrence versus temperature for several fixed values of the exchange anisotropy, taking $J=0.02$ and $B=0.6$. (**c**) The same function against magnetic field for various values of the exchange anisotropy at low temperature $T=0.02$ and fixed $J=0.02$. (**d**) The exchange coupling dependence of the concurrence at low temperature $T=0.02$ and fixed $\Delta =1$, where different values of the magnetic field are assumed.

## Results and discussion

To get an insight into the pairwise entanglement, we have plotted the concurrence (5) as a function of the temperature *T* for several values of the magnetic field *B* and fixed $\Delta =1$ as well as $J=0.02$ in Fig. 2a. It is observed that the concurrence is a decreasing function of *T* from its maximum value $C(\rho _T )=1$ for weak values of *B*, but its behavior is different for stronger magnetic fields. Besides, it undergoes substantial changes upon the anisotropy alterations, as seen in Fig. 2b. As a matter of fact, an increase in the $\Delta$ strengthens the entanglement. The field dependence of the concurrence is depicted in Fig. 2c at low temperature $T=0.02$ and low coupling constant $J=0.02$, where several fixed values of the $\Delta$ are considered. One sees that for the finite values of parameters *B*, *J*, and *T*, the concurrence does not reach its maximum as soon as the anisotropy $\Delta$ enhances from zero. For $\Delta >0$, the concurrence remains steady at low magnetic fields, then sharply increases nearby the critical magnetic field and reaches its maximum instantly. Eventually, this quantity shows a steep decrease close to the second critical magnetic field. Fig. 2d displays the concurrence against the exchange coupling *J* at low temperature $T=0.02$ and $\Delta =1$ for various fixed values of the magnetic field. Here, it is evident that the concurrence has an anomalous behavior in the vicinity of some critical points. A sudden death of this measure of entanglement happens nearby the critical point $J=-1$.

**Figure 3:**
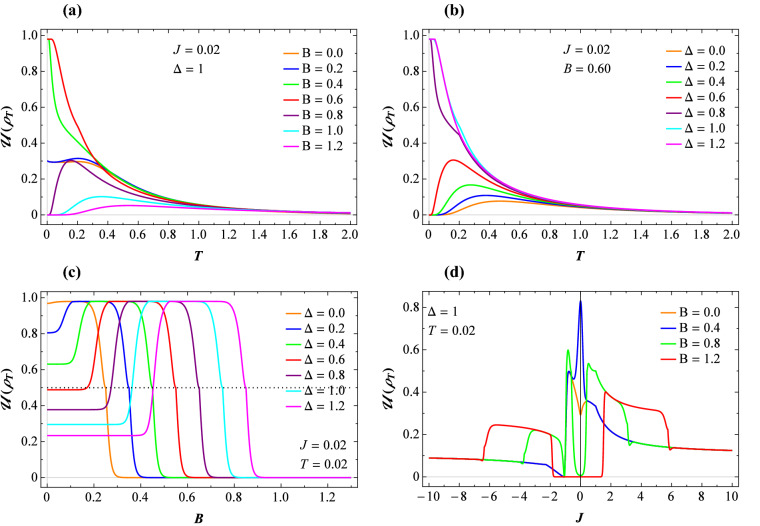
LQU with respect to the same parameter sets in Fig. 2.

Figure 3 illustrates the thermal LQU (16) of the model in different planes. Two plots (a) and (b) depict the temperature dependence of this function for fixed $J=0.02$, such that Fig. 3a corresponds to the several values of the magnetic field and $\Delta =1$, whereas the other one corresponds to the case when several values of the anisotropy are assumed at fixed $B=0.6$. Some differences are evident between LQU and concurrence. For example, the latter reaches its maximum value of 1 at finite low temperatures while the former, under the same conditions, does not. In addition, the concurrence vanishes at a critical temperature, while upon heating, LQU tends to zero but does not vanish. This particular behavior indicates the robustness of LQU against the concurrence at higher temperatures. Fig. 3c displays the LQU as a function of the magnetic field for several values of the exchange anisotropy, supposing low temperature $T=0.02$ and $J=0.02$. With the increase of the magnetic field, this function sharply decreases and ultimately vanishes nearby the same critical magnetic point to the concurrence. It is worth mentioning that another notable difference between the magnetic behavior of LQU and concurrence is that, the former behaves anomalously when drops down in height to $\mathcal {U}(\rho _T )=1/2$. This phenomenon indicated by a horizontal dotted line, however, it was not observed in the concurrence behavior. This is in fact the well-known sudden change behavior for the discord-like quantum correlations, which is caused by the optimization procedure in their respective definitions^75,76^. As a result, the LQU is more sensitive than the concurrence to demonstrate thermal fluctuations or discontinuous phase spectra.

Last but not least, let us also emphasize another important consequence of the LQU analysis with respect to the coupling constant *J*. Fig. 3d illustrates the LQU versus the coupling constant *J* at low temperature $T=0.02$ and $\Delta =1$, where four different values of the magnetic field have been taken. For mixed ferromagnetic-antiferromagnetic case $J<0$, we see that this function does not vanish but tends to $\mathcal {U}(\rho _T )=0.1$ as *J* decreases further than critical point $J=-1$. On the other hand, for the ferromagnetic coupling $J>0$, the LQU falls down close to the critical exchange and it shows a discontinuous alteration at low magnetic field $B=0.4$ (blue line). Hence, the LQU could be a good witness of the discontinuous thermal behaviors of the model that is more efficient than the concurrence in this medium.

**Figure 4:**
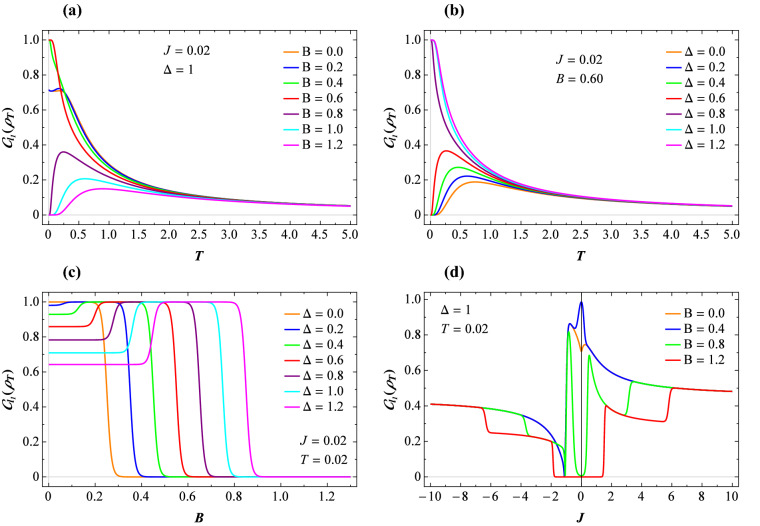
Quantum coherence with respect to the same parameter sets in Figs. 2 and 3.

We depict typical thermal variations of the $l_{1}-$norm of coherence of our model in Fig. 4. Plots (a) and (b) manifest the temperature dependence of the quantum coherence for the fixed $J=0.02$ and various values of other parameters. It is evident that $C_{l_{1}}(\rho _T )$ experiences its maximum at low temperature such as the concurrence. On the other hand, when the temperature increases, at low magnetic fields and low anisotropies such a function gradually decreases and tends to zero but does not vanish neither at higher magnetic fields nor for stronger exchange anisotropies. Returning to Fig. 3, it is observable that the $l_{1}-$ norm of coherence and LQU have somehow similar behavior to each other at high temperatures.

Figure 4c displays the coherence as a function of the magnetic field at low temperature $T=0.02$ and low exchange coupling $J=0.02$, where several fixed values of the anisotropy $\Delta$ are assumed. Compared to previous figures, the same behavior to the coherence is evident close to the critical magnetic fields. The $l_{1}-$norm of coherence $C_{l_{1}}$ versus the coupling constant *J* at low temperature is plotted in Fig. 4d, where fixed value $\Delta =1$ and four different magnetic fields are selected. It is visible a quite different behavior of this function compared with the LQU and the concurrence. Namely, for the case when $J<0$, by decreasing the exchange coupling *J* further than the critical point $J=-1$, the quantum coherence not only does not vanish but also increases notably. Generally speaking, one can deduce from our observations that the $l_{1}-$ norm of coherence is more efficient than both concurrence and LQU to predict the quantumness of the thermal state even at high temperatures and high magnetic fields.

### Quantum teleportation

In this section, we study quantum teleportation for an entangled mixed state as a resource, acts as a generalized depolarizing channel^77–79^. Next, we investigate the effects of the anisotropy and the magnetic field on the possibility of teleportation through the model under verification. Let us assume the input state being an arbitrary unknown two-qubit pure state $\left| \psi _\text {in}\right\rangle$, such as

19 $$\begin{aligned} \left| \psi _\text {in}\right\rangle =\cos (\theta /2)\left| 10\right\rangle +e^{i\phi }\sin (\theta /2)\left| 01\right\rangle , \quad \forall \quad 0\le \theta \le \pi , \quad 0\le \phi \le 2\pi . \end{aligned}$$

From the mathematical point of view, the quantum channel is known as a completely positive and trace-preserving operator. Via this mechanism, an input density operator is mapped to an output density operator^77^. Generally, when the quantum state is teleported through the mixed channel $\mathcal {\rho }_\text {ch}$, the output replica state $\mathcal {\rho }_\text {out}$ can be obtained by applying joint measurements and the local unitary transformations on the input state $\rho _\text {in}=\left| \psi _\text {in }\right\rangle \left\langle \psi _\text {in}\right|$, hence

20 $$\begin{aligned} \mathcal {\rho }_\text {out}=\sum _{i,j\in \{0,x,y,z\}}p_{i}p_{j}(\sigma ^{i}\otimes \sigma ^{j})\rho _\text {in}(\sigma ^{i}\otimes \sigma ^{j}), \end{aligned}$$

where $\sigma _{0}=I$ (*I* is the $2\times 2$ identity matrix) and $p_{i}= \mathrm {Tr}$($E^{i}\mathcal {\rho }_\text {ch}$) satisfies the condition $\sum _{i}p_{i}=1$. $E^{0}=\left| \Psi ^{-}\right\rangle \left\langle \Psi ^{-}\right| ,$ $E^{1}=\left| \Phi ^{-}\right\rangle \left\langle \Phi ^{-}\right| ,$ $E^{2}=\left| \Psi ^{+}\right\rangle \left\langle \Psi ^{+}\right| ,$ and $E^{3}=\left| \Phi ^{+}\right\rangle \left\langle \Phi ^{+}\right| ,$ from which $\{\left| \Psi ^{\pm }\right\rangle ,$ $\left| \Phi ^{\pm }\right\rangle \}$ stand for the well known Bell states. In this paper, we consider the quantum channel as $\mathcal {\rho }_\text {ch}=\rho _T$. Therefore, The output density operator $\mathcal {\rho }_\text {out}$ takes the form

21 $$\begin{aligned} \mathcal {\rho }_\text {out}= \begin{pmatrix} \widetilde{\mathcal {\rho }}_{11} &{} . &{} . &{} . \\ . &{} \widetilde{\mathcal {\rho }}_{22} &{} \widetilde{\mathcal {\rho }}_{23} &{} . \\ . &{} \widetilde{\mathcal {\varrho }}_{23} &{} \widetilde{\mathcal {\rho }}_{33} &{} . \\ . &{} . &{} . &{} \widetilde{\mathcal {\rho }}_{11} \end{pmatrix} , \end{aligned}$$

where

22 $$\begin{aligned} \widetilde{\mathcal {\rho }}_{11}= & {} (\mathcal {\rho }_{11}+\mathcal {\rho } _{44})(\mathcal {\rho }_{22}+\mathcal {\rho }_{33}), \nonumber \\ \widetilde{\mathcal {\rho }}_{22}= & {} (\mathcal {\rho }_{11}+\mathcal {\rho } _{44})^{2}\cos ^{2}(\theta /2)+(\mathcal {\rho }_{22}+\mathcal {\rho } _{33})^{2}\sin ^{2}(\theta /2), \nonumber \\ \widetilde{\mathcal {\rho }}_{33}= & {} (\mathcal {\rho }_{11}+\mathcal {\rho } _{44})^{2}\sin ^{2}(\theta /2)+(\mathcal {\rho }_{22}+\mathcal {\rho } _{33})^{2}\cos ^{2}(\theta /2), \nonumber \\ \widetilde{\mathcal {\rho }}_{23}= & {} 2e^{i\phi }\mathcal {\rho }_{23}^{2}\sin \theta . \end{aligned}$$

To describe the quality of the process of teleportation, it is often quite useful to study the fidelity between $\rho _\text {in}$ and ${\rho }_\text {out }$ to characterize the teleported state. When the input state is a pure state, one can apply the concept of fidelity as a useful indicator of the teleportation performance of a quantum channel quantifier^80,81^. The fidelity is defined as^6^

23 $$\begin{aligned} \mathscr {F}=\left( \mathrm {Tr}\sqrt{\sqrt{\rho _\text {in}}\mathcal {\rho }_ \text {out}\sqrt{\rho _\text {in}}}\right) ^{2}. \end{aligned}$$

The fidelity is near zero if the input and output states are orthogonal, which means the information is fully destroyed during the transmission process, so the teleportation fails. While it is close to unity, it signifies that the input state is identical to the output state. In the situation when $0<\mathscr {F}<1$, the quantum information is subjected to distortions after transmitting to some extent. Through a straightforward calculation for our case, one finds

24 $$\begin{aligned} \mathscr {F}=\frac{\sin ^{2}\theta }{2}[(\mathcal {\rho }_{11}+\mathcal {\rho } _{44})^{2}+4\mathcal {\rho }_{23}^{2}-(\mathcal {\rho }_{22}+\mathcal {\rho }_{33})^{2}]+(\mathcal {\rho }_{22}+\mathcal {\rho }_{33})^{2}. \end{aligned}$$

The average fidelity of teleportation $\mathscr {F}_{A}$ can be formulated as^82,83^

25 $$\begin{aligned} \mathscr {F}_{A}=\frac{1}{4\pi }\int _{0}^{2\pi }d\phi \int _{0}^{\pi }d\theta \mathscr {F}\sin \theta , \end{aligned}$$

by integrating Eq. (25), the average fidelity $\mathscr {F}_{A}$ for our case can be thus expressed as

26 $$\begin{aligned} \mathscr {F}_{A}=\frac{1}{3}[(\mathcal {\rho }_{11}+\mathcal {\rho }_{44})^{2}+4 \mathcal {\rho }_{23}^{2}+2(\mathcal {\rho }_{22}+\mathcal {\rho }_{33})^{2}]. \end{aligned}$$

**Figure 5:**
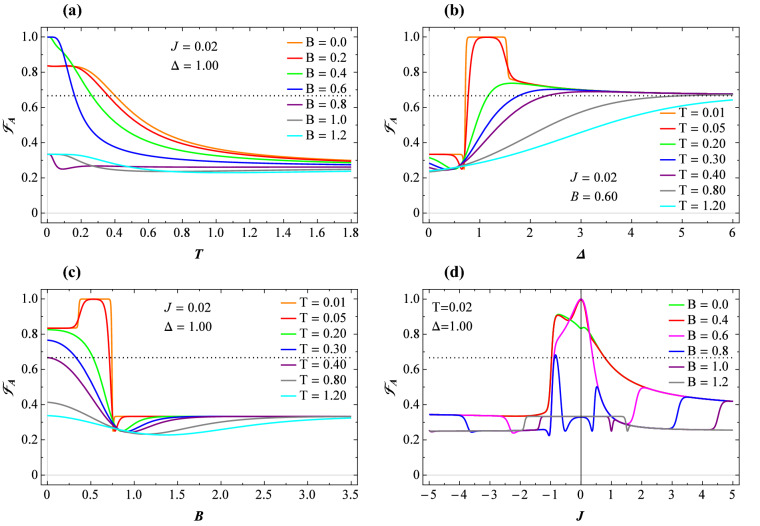
(**a**) The average fidelity $\mathscr {F}_{A}$ versus temperature, assuming $J=0.02$, $\Delta =1$, and several fixed values of the magnetic field. (**b**) $\mathscr {F}_{A}$ as a function of anisotropy $\Delta$ for fixed $J=0.02$, $B=0.6$, and different values of the temperature. (**c**) Field dependence of $\mathscr {F}_{A}$ for various temperatures, where other parameters have been taken as $J=0.02$ and $\Delta =1$. (**d**) the same function at low temperature $T=0.02$ against the coupling constant *J* when different fixed values of the magnetic field are considered such that $\Delta =1$. Dotted lines indicate the classical limit of fidelity 2/3.

It is obvious that the average fidelity $\mathscr {F}_{A}$ depends on the quantum channel (thermal state) parameters in this case. In order to transmit a quantum state better than the classical communication protocols, $\mathscr {F}_{A}$ must be greater than $\frac{2}{3}$ which is the best fidelity in the classical world. In Fig. 5a, the average fidelity as a function of the temperature for the weak coupling $J=0.02$ and fixed $\Delta =1$ is shown, where several fixed values of the magnetic field have been assumed. It is clear from this plot, that $\mathscr {F}_{A}$ does not reach the limit of quantum fidelities for $B\gtrsim 0.8$, hence the teleportation of information happens for the magnetic field rage $B<0.8$. An increase in the temperature leads to a decrease in the possibility of teleportation. With looking to Figs. 2a, 3a and 4a, one realizes that for the parameter sets that situation $\mathscr {F}_{A}<2/3$ occurs, three functions, i.e., concurrence, LQU, and coherence show a sharp decrease until they vanish at low temperatures and never reach their maximum values under cooling condition. On the other hand, in the parameter regions which $\mathscr {F}_{A}>2/3$, all three functions gradually tend towards maximum value under cooling.

In Fig. 5b, we depict the average fidelity versus the anisotropy $\Delta$ for several fixed temperatures and parameter sets $J=0.02$ and $B=0.6$. A reentrance point is evident in this plot at which a sharp change in the average fidelity behavior happens and this quantity immediately reaches its maximum $\mathscr {F}_{A}=1$ within the anisotropy interval $0.8\lesssim \Delta \lesssim 1.5$ at extremely low temperatures, accompanying with the achieving maximum value of the concurrence, LQU, and coherence. Generally, the average fidelity tends to limit value 2/3 with increasing the anisotropy. The relevant field dependence of the average fidelity at $J=0.02$ and $\Delta =1$ shown in Fig. 5c has also similar behavior. It can be seen from this plot a steep decrease as well as line accumulation in the average fidelity function at low temperatures regime in the vicinity of critical magnetic field. Under heating, $\mathscr {F}_{A}$ decreases at low magnetic fields. Accordingly, the possibility of teleportation through this model is restricted.

The most interesting finding from the fidelity investigations is manifested in Fig. 5d by which we illustrate the average fidelity with respect to the exchange coupling *J* at low temperature $T=0.02$ and $\Delta =1$. Similar to the other three functions, $\mathscr {F}_{A}$ anomalously behaves nearby the critical exchange couplings. Surprisingly, in the antiferromagnetic region of *J*, close to the critical point $J=-1$, the average fidelity sharply drops to its minimum. Consequently, the average fidelity could be an eligible candidate to trace the thermal fluctuations of a typical Heisenberg spin-1/2 square compound possessing either ferromagnetic or mixed ferromagnetic-antiferromagnetic exchange couplings.

**Figure 6:**
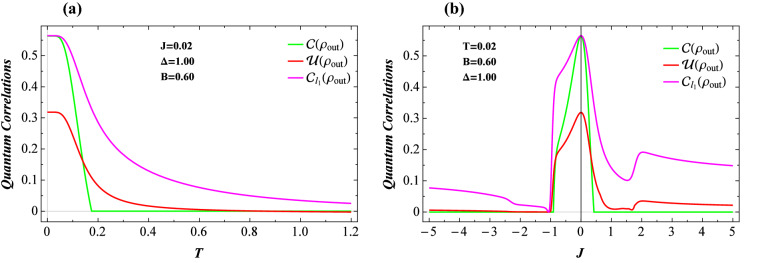
(**a**) Three quantum criteria for the output state versus temperature *T* and parameter sets $J=0.02$, $\Delta =1$, and $B=0.6$. (**b**) The same quantifiers as functions of the coupling constant *J* at low temperature $T=0.02$, assuming $\Delta =1$ and $B=0.6$.

Finally, we present our results concerning with the temperature dependence of three discussed quantities for the output state $\rho _\text {out}$, i.e., $C(\rho _\text {out})$, $\mathscr {U}(\rho _\text {out})$, and $C_{l_1}(\rho _\text {out})$ for fixed $J=0.02$, $\Delta =1$, and $B=0.6$ in Fig. 6a. All functions decrease from a typical maximum when the temperature increases. The concurrence suddenly vanishes at a critical temperature, while other two functions monotonically decreases. The notable difference between $\mathscr {U}(\rho _ \text {out})$ and $C_{l_1}(\rho _\text {out})$ is that, the former vanishes at a specific critical temperature while the latter does not reach zero even at high temperatures. Moreover, we plot in Fig. 6b the aforementioned quantities as functions of the exchange coupling *J* at low temperature $T=0.02$ and fixed $B=0.6$ and $\Delta =1$. By assuming mixed ferromagnetic-antiferromagnetic case ($J<0$), all three functions sharply decrease and vanish nearby the critical point $J=-1$, reminding the same thermal behaviors of these criteria as well as fidelity at this point. With decrease of the *J* further than $J=-1$, the concurrence remains zero, while the LQU and quantum coherence arise from zero and reach the non-zero values. On the other side, within the ferromagnetic region ($J>0$), one can observe the entanglement sudden death at $J\approx 0.5$, whereas the LQU and coherence show a sudden decline at this point, but remain alive even for higher values of *J*. According to our observations, we generally claim that the $l_{1}-$norm of coherence could reveal more quantum information about the system under study, which is consistent with previous results.

## Concluding remarks and outlook

We have considered a four-qubit cluster complex on a spin-1/2 Heisenberg XXZ model involving an exchange anisotropy in the presence of a magnetic field along the *z*-axis. Both pure ferromagnetic and mixed ferromagnetic-antiferromagnetic exchange couplings between nearest-neighbor spins have been verified. We then studied three quantum criteria such as concurrence, local quantum uncertainty, and quantum coherence for a pair of spins. Consequently, we have demonstrated that all three functions behave anomalously close to the critical points. At low temperature and weak coupling constant, we could observe maximum entanglement between selected pair of spins. According to our observations, we convincingly concluded that the quantum coherence is generally more sensitive than the other ones to witnessing the thermal fluctuations in different Hamiltonian parameter sets. We also understood that by tuning the strength of the anisotropy parameter, a significant enhancement on the entanglement and various thermal non-classical correlations and coherence can be achieved. Finally, we have examined the possibility of teleportation through the model under consideration. We found that within a special interval of assumed exchange anisotropy, the average fidelity significantly enhances. However, the average fidelity tends to limit value 2/3 with further increase of the anisotropy. The average fidelity also represented different behaviors such as line accumulation and sharp dropping nearby the critical points.

Investigating aforementioned quantum correlations quantifiers, coherence, and the fidelity of teleportation for similar classes of small spin clusters might enlighten the quantum nature of them that would be applicable in different subjects such as quantum information processing, quantum communication, and spintronics. Furthermore, we think that our model can pleasantly bring insight into the ground-state phase diagram and several important magnetic and quantum features of some real materials with similar square-shaped structures. Our future activity will concern with this direction.

## Methods

### Eigenvalues and eigenstates of the Hamiltonian

In this section, we give the eigenvalues and the corresponding eigenstates of Hamiltonian (1) in terms of the standard computational basis $\mathscr{B}=\left\{ \vert 000 \rangle ,\vert 001 \rangle ,\vert 010 \rangle ,\vert 011 \rangle ,\vert 100 \rangle ,\vert 101 \rangle ,\vert 110 \rangle ,\vert 111 \rangle \right\}$. The eigenvalues of the Hamiltonian are

27 $$\begin{aligned} \begin{array}{l} E_{1}=-2B-\frac{1}{4}J-\frac{1}{4}+\frac{1}{4}\sqrt{K}, \quad E_{2}=-2B-\frac{1}{4}J-\frac{1}{4}-\frac{1}{4}\sqrt{K}, \quad E_{3}=E_{4}=0, \\ E_{5}=2B-\frac{1}{4}J-\frac{1}{4}+\frac{1}{4}\sqrt{K}, \quad E_{6}=2B-\frac{1}{4}J-\frac{1}{4}-\frac{1}{4}\sqrt{K}, \\ E_{7}=2B+\frac{1}{4}J+\frac{1}{4}+\frac{1}{4}\sqrt{K}, \quad E_{8}=2B+\frac{1}{4}J+\frac{1}{4}-\frac{1}{4}\sqrt{K}, \\ E_{9}=\Delta -4B, \quad E_{10}=-2B+\frac{1}{4}J+\frac{1}{4}+\frac{1}{4}\sqrt{K}, \\ E_{11}=-2B+\frac{1}{4}J+\frac{1}{4}-\frac{1}{4}\sqrt{K}, \quad E_{12}=\Delta +4B, \\ E_{13}=-\frac{1}{2}\Delta +\frac{1}{2}\sqrt{\Delta ^{2}+J-2J+1}, \quad E_{14}=-\frac{1}{2}\Delta -\frac{1}{2}\sqrt{\Delta ^{2}+J^{2}-2J+1}, \\ E_{15}=-\frac{1}{2}\Delta +\frac{1}{2}\sqrt{\Delta ^{2}+5J^{2}+2J+1}, \quad E_{16}=-\frac{1}{2}\Delta -\frac{1}{2}\sqrt{\Delta ^{2}+5J^{2}+2J+1}, \end{array} \end{aligned}$$

with $K=5J^{2}-2J+1$. The corresponding eigenstates are given, respectively, by

28 $$\begin{aligned} \begin{array}{l} \left| \psi _{1}\right\rangle =a_{+}\left| 0001\right\rangle +b_{+}\left| 0010\right\rangle +\left| 0100\right\rangle +\left| 1000\right\rangle , \\ \left| \psi _{2}\right\rangle =a_{-}\left| 0001\right\rangle +b_{-}\left| 0010\right\rangle +\left| 0100\right\rangle +\left| 1000\right\rangle , \\ \left| \psi _{3}\right\rangle =-\left| 0011\right\rangle +\left| 1100\right\rangle , \\ \left| \psi _{4}\right\rangle =\alpha \left| 0011\right\rangle +\left| 0101\right\rangle +\left| 1010\right\rangle , \\ \left| \psi _{5}\right\rangle =c_{+}\left| 0111\right\rangle +d_{+}\left| 1011\right\rangle +\left| 1101\right\rangle +\left| 1110\right\rangle , \\ \left| \psi _{6}\right\rangle =c_{-}\left| 0111\right\rangle +d_{-}\left| 1011\right\rangle +\left| 1101\right\rangle +\left| 1110\right\rangle , \\ \left| \psi _{7}\right\rangle =c_{-}\left| 0111\right\rangle -d_{-}\left| 1011\right\rangle -\left| 1101\right\rangle +\left| 1110\right\rangle , \\ \left| \psi _{8}\right\rangle =c_{+}\left| 0111\right\rangle -d_{+}\left| 1011\right\rangle -\left| 1101\right\rangle +\left| 1110\right\rangle , \\ \left| \psi _{9}\right\rangle =\left| 0000\right\rangle , \\ \left| \psi _{10}\right\rangle =a_{-}\left| 0001\right\rangle -b_{-}\left| 0010\right\rangle -\left| 0100\right\rangle +\left| 1000\right\rangle , \\ \left| \psi _{11}\right\rangle =a_{+}\left| 0001\right\rangle -b_{+}\left| 0010\right\rangle -\left| 0100\right\rangle +\left| 1000\right\rangle , \\ \left| \psi _{12}\right\rangle =\left| 1111\right\rangle , \\ \left| \psi _{13}\right\rangle =-\left| 0101\right\rangle +e_{+}\left| 0110\right\rangle -e_{+}\left| 1001\right\rangle +\left| 1010\right\rangle , \\ \left| \psi _{14}\right\rangle =-\left| 0101\right\rangle +e_{-}\left| 0110\right\rangle -e_{-}\left| 1001\right\rangle +\left| 1010\right\rangle , \\ \left| \psi _{15}\right\rangle =\left| 0011\right\rangle +\beta \left| 0101\right\rangle +f_{+}\left| 0110\right\rangle +f_{+}\left| 1001\right\rangle +\beta \left| 1010\right\rangle +\left| 1100\right\rangle , \\ \left| \psi _{16}\right\rangle =\left| 0011\right\rangle +\beta \left| 0101\right\rangle +f_{-}\left| 0110\right\rangle +f_{-}\left| 1001\right\rangle +\beta \left| 1010\right\rangle +\left| 1100\right\rangle , \end{array} \end{aligned}$$

where the following notations are adopted

29 $$\begin{aligned} \begin{array}{l} a_{\pm }=\dfrac{2J\big (3J^{2}\mp J\sqrt{K}-2J\pm \sqrt{K}-1\big )}{\big ( 2J^{2}+J\pm \sqrt{K}-1\big )\big (J+1\mp \sqrt{K}+1\big )}, \quad \alpha =-\dfrac{J+1}{ J}, { \ \ }\beta =\dfrac{1}{2}\dfrac{J+1}{J},\\ b_{\pm }=\dfrac{J\big (J-3\pm \sqrt{K}\big )}{\big (2J^{2}+J\pm \sqrt{K}-1\big )} , \quad c_{\pm }=\dfrac{2J^{2}\big (J^{2}\mp J\sqrt{K}+2J\pm \sqrt{K}-3\big )}{\big ( J^{3}\mp (J^{2}\pm J\pm 1)\sqrt{K}+1\big )\big (J+1\mp \sqrt{K}+1\big )}, \\ d_{\pm }=\dfrac{J(2J^{2}-3J\mp \sqrt{K}-1)}{\big (J^{3}\mp (J^{2}\pm J\pm 1) \sqrt{K}+1\big )}, \quad e_{\pm }=\dfrac{J-1}{\big (\Delta \pm \sqrt{\Delta ^{2}+J^{2}-2J+1}\big )}, \\ f_{\pm }=-\dfrac{1}{2}\dfrac{5J^{2}+2J+1}{J\big (\Delta \pm \sqrt{\Delta ^{2}+5J^{2}+2J+1}\big )}. \end{array} \end{aligned}$$

## Data Availability

All data generated or analyzed during this study are included in this paper.
